# Calcium Channel Blocker-Induced Thrombocytopenia in the Intensive Care Unit: A Rare Presentation and Review of the Literature

**DOI:** 10.7759/cureus.42918

**Published:** 2023-08-03

**Authors:** Allen Zhou, Michael Sandhu, Brian Min, Gaston Habib, Markus Gutsche

**Affiliations:** 1 Internal Medicine, State University of New York Upstate Medical University, Syracuse, USA; 2 Nephrology, State University of New York Upstate Medical University, Syracuse, USA; 3 Pulmonology and Critical Care, State University of New York Upstate Medical University, Syracuse, USA

**Keywords:** general pharmacology, drug induced thrombo, medical intensive care unit (micu), clinical hematology, toxicology and poisoning

## Abstract

Patients with thrombocytopenia may report easy bruising, abnormal bleeding, and fatigue. Drug-induced thrombocytopenia has been reported with a variety of medications, most commonly heparin products, sulphonamides, carbamazepine, nonsteroidal anti-inflammatory drugs, anti-epileptic drugs, and chemotherapy. We present the case of a 58-year-old female with severe thrombocytopenia attributed to a calcium channel blocker (CCB) overdose, a very rare cause of thrombocytopenia. We discuss the diagnostic work-up and management in the intensive care unit and perform a literature review.

## Introduction

Thrombocytopenia is a rare and fatal side effect triggered by certain medications. Medications that are used in the hospital that may lead to thrombocytopenia include heparin, antibiotics, and pain medications [1]. However, studies have shown that these medications are more likely to cause thrombocytopenia when taken in excess, as opposed to recommended doses [2]. Other common etiologies for thrombocytopenia in the hospital include infections (such as human immunodeficiency virus or hepatitis C), nutritional deficiencies, chronic alcohol use, and autoimmune diseases. Calcium channel blockers (CCBs) are a class of medication used in the management of hypertension and have been reported to cause thrombocytopenia in three other review articles [3,4]. Nifedipine was implicated in one and amlodipine in two other case reports, with anti-amlodipine antibodies confirmed in one of the cases [5,6]. However, these cases included patients taking CCBs at therapeutic doses. We are reporting a case of amlodipine-induced severe thrombocytopenia from a drug overdose.

## Case presentation

A 58-year-old Caucasian woman with a history of hyperlipidemia, hypertension, generalized anxiety disorder, and recent right parietal hemorrhage six weeks prior to her admission presented after a suicide attempt by overdosing with amlodipine, metoprolol, and alprazolam. Two days prior to her suicide attempt, she refilled a 30-day prescription of amlodipine 10 mg PO every day. She was found unconscious by her roommate. She received calcium gluconate and glucagon and was managed with a transcutaneous pacer for severe sinus bradycardia with cardiogenic shock. On admission, she developed severe transaminitis. It returned to a mildly elevated state of transaminitis after the second day in the ICU and fluctuated between 60-90 Units/L afterward.

She required hemodynamic pressor support on arrival with an initial blood pressure of 75/60 mmHg in the form of norepinephrine, epinephrine, and dobutamine and was treated with continuous insulin infusion and methylene blue. The transcutaneous pacer was successfully replaced with a transvenous pacer.

Her course was further complicated by oliguric acute kidney injury (with creatinine levels of 2.7 mg/dL from a baseline of 1.1 mg/dL) and lactic acidosis that nephrology managed with continuous veno-venous hemofiltration (CVVH), sodium bicarbonate, and phosphate infusions. The patient was found to have severe thrombocytopenia with a decline in her platelet count from 200,000 cells/uL to 7,000 cells/uL roughly 48 hours after her overdose. Fibrinogen level, Coomb’s test, HLA autoantibodies, and heparin IgG were normal. Schistocytes were absent on the peripheral blood smear. Her hemoglobin and white blood count stayed within normal limits. Her hemoglobin did not experience a similar crash and was stable between 10-12 mg/dL and gradually dwindled during her hospital course. She had leukocytosis which continued to elevate during her entire stay in the MICU.

**Figure 1:**
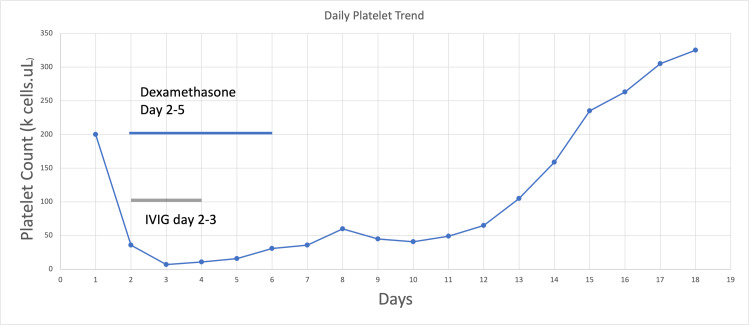
Platelet Trend With Days Following Overdose

The patient was managed with platelet transfusions and 50 grams of intravenous immunoglobulin (IVIG) (~1 gram/kg) during Days 2-3 of her hospital stay in addition to 40 mg IV QD of dexamethasone from Days 2-5. Eventually, the patient’s platelet count began to rebound after reaching a nadir of 7,000 cells/uL. Eventually, following IVIG and dexamethasone the platelet count rose to 50,000 cells/uL and stabilized in the 60,000-80,000 cells/uL range approximately seven to eight days from the estimated time of the amlodipine overdose (Figure 1).

Unfortunately, given her recent intracerebral hemorrhagic stroke, the patient experienced renewed bleeding, most likely caused by her severe thrombocytopenia, and remained critically ill with poor mentation and inability to wean off mechanical ventilation. At the same time, her course was further complicated by ventilator-associated pneumonia (VAP) with methicillin-sensitive staphylococcus aureus (MSSA) and Klebsiella, as well as multi-organ failure including acute persistent anuric renal failure requiring CVVH. Eventually, her decision-maker transitioned her care to focus on palliative measures with compassionate extubation, passing away shortly thereafter.

**Figure 2:**
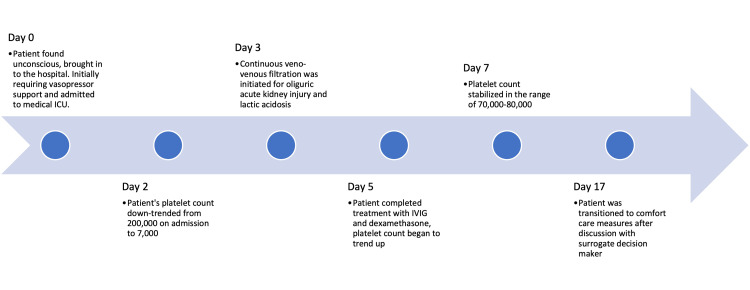
Timeline of Events During the Patient's Hospital Course

## Discussion

Thrombocytopenia is a common finding in critically unwell patients with a broad differential including disseminated intravascular coagulopathy and idiopathic thrombolytic purpura. Our patient developed severe thrombocytopenia after overdosing on amlodipine, metoprolol, and alprazolam. Amlodipine has a half-life of 48 hours [7]. In our case, the onset of thrombocytopenia after oral overdose ingestion was approximately 48 hours with a nadir of 7,000 cells/uL during Day 3 of hospitalization.

Platelet suppression or destruction of platelets are proposed mechanisms of drug-induced thrombocytopenia (DITP). In vitro studies have shown that verapamil and diltiazem - and dihydropyridine calcium blockers to a lesser extent - downregulate platelet aggregation via inhibition of alpha 2 adrenergic receptors. CCBs have also been shown to decrease the concentration of thromboxane B2, the inactive metabolite of thromboxane A2, an eicosanoid that has thrombopoietic and thrombotic properties [6]. Rostagno et al. concluded that there would need to be a complete inhibition of the cyclooxygenase pathway to completely inhibit the activation of platelets with thromboxane A2 and that the mild decrease in thromboxane activity was negligible [6]. However, our patient had recently filled a prescription with 60 tablets of amlodipine 5 mg and possibly ingested a cumulative dose of 300 mg of amlodipine. We postulate that this dose may have had a significant effect on platelet inhibition and degradation via inhibition of thromboxane A2 production and the cyclooxygenase pathway. In animal models, CCBs upregulate prostacyclin production in dogs, downregulating platelet activity. Dihydropyridine calcium blockers have been shown to decrease platelet aggregation in dogs with vascular grafts [8]. Furthermore, Garbe et al. demonstrated that amlodipine triggered the production of drug-dependent antibodies against platelets [5]. Autoimmune thrombocytopenia due to medications is underdiagnosed because it may be confounded with idiopathic thrombocytopenia. The pathophysiology is unclear, but it has been proposed that the sensitizing drug augments the binding affinity of the immunoglobulins to platelets. However, the binding affinity is insignificant without medication. Alternatively, the antibodies and medication may create conformational changes in platelet surface proteins [9]. 

We performed a comprehensive literature review to find three case reports of calcium channel blocker-induced thrombocytopenia after long-term use. Nifedipine was associated with thrombocytopenia in one patient and amlodipine was the causative agent in two others. The dihydropyridine class of calcium channel blockers has been shown to downregulate platelet aggregation. In previous case reports, the patients were using amlodipine either chronically or acutely. In one case, a 79-year-old male who had been on long-term amlodipine began to suddenly develop hematomas, hematuria, and petechiae [5]. After holding amlodipine for seven days and receiving five days of steroids, the patient’s platelets rebounded from 1,000 cells/uL to 201,000 cells/uL. It was possible to identify the amlodipine as the causative agent after the thrombocytopenia recurred on restarting amlodipine with confirmation of antibodies directed against platelets [5]. Another case report presented a patient with thrombocytopenia after two to three days of amlodipine consumption who had diffuse petechiae and profuse, frequent menorrhagia at a nadir platelet level of 12,000 cells/uL. Removal of amlodipine for seven days improved the platelet count to 172,000 cells/uL [10]. There has been one documented case report of thrombocytopenia due to nifedipine that caused the platelet level to drop to 20,000 cells/uL after 20 days on nifedipine. Platelets returned to baseline after holding nifedipine for 10 days. 

Another patient developed DITP from dual usage of simvastatin and amlodipine; she presented with multiple ecchymoses on arrival at the emergency department. Following 17 days of hospitalization prior to returning and seven days of prednisolone (1 mg/kg), her platelet count normalized. Drug-induced thrombocytopenia was confirmed after restarting simvastatin; the patient had a platelet count of 98,000 cells/uL and subsequent hives. Afterward, she mistakenly took amlodipine 10 mg once dropping her platelet to 32,000 cells/uL. She was asymptomatic, and no further intervention was warranted [11]. 

Amlodipine is a rare cause of DITP. Consequently, DITP may be attributed to immune thrombocytopenic purpura (ITP) [7]. Drug-induced thrombocytopenia (DITP) appears suddenly and is often severe, with a risk of major bleeding and death [2]. DITP can develop either from dose-dependent bone marrow suppression or from peripheral platelet destruction [2]. ITP is thrombocytopenia not related to a systemic disease, whereas DITP involves drug ingestion [3]. Early recognition of DITP is essential, especially in critically ill patients, to promptly remove the offending agent [3]. Platelet counts usually recover within 1-10 days after discontinuation of the offending agent [4]. Our patient’s platelet count remained below 150,000 cells/uL until Days 12-13 of hospitalization, conceivably due to an overdose from amlodipine contrasting consumption of a therapeutic dose.

Amlodipine has a half-life of 24-48 hours [11,12]. The main course of treatment for DITP is steroids and sometimes IVIG [8]. In our patient, the platelet count started to reach normal levels approximately 13 days after her overdose at 159,000 cells/uL. While the IVIG and the corticosteroids stabilized the platelet count between 70,000-85,000 cells/uL, it did not fully recover to normal levels immediately. There may have been confounding variables by other causes such as multi-organ failure - namely transaminitis and bone marrow suppression from cardiogenic shock and sepsis from ventilator-associated pneumonia. However, her thrombocytopenia is likely due to transaminitis or septic shock because her liver functions stayed mildly elevated after the severe transaminitis during the first 48 hours after admission. Bone marrow suppression is less likely because the patient’s hemoglobin dwindled gradually during her three weeks in the ICU, contrasting to the sudden crash of platelets from 200,000 cells/uL to 7,000 cells/uL within 24 hours; this gradual anemia is more likely explained from the extensive blood draws for continuous monitoring in the ICU. Furthermore, her white blood count had always stayed elevated during her hospital stay. Septic shock was much less likely because the patient developed ventilator-associated pneumonia during Day 10 of hospitalization. Despite her infection, the patient’s platelet had a continuous upward trend and recovered to normal limits by Day 12, making sepsis less likely. Lastly, this patient never had pancytopenia during her hospital stay which would be consistent with organ failure, bone marrow suppression, or shock.

The patient had progressive bleeding at a previous intraparenchymal hemorrhage that occurred six weeks prior to her overdose. During that admission, the patient initially presented with left-sided weakness and paresthesias. Her Glasgow Coma Scale (GCS) score was 15. She was admitted and a repeat MRI of the brain showed stability. The patient's symptoms improved prior to her admission to our institution. Interestingly, our patient did not manifest clear signs of bleeding - hematomas, menorrhagia, petechiae, and ecchymoses - during her physical exam, which contrasted with the findings of previous case reports.

## Conclusions

To our knowledge, we present the first case of severe thrombocytopenia after an overdose with amlodipine wherein the duration of the thrombocytopenia was longer likely in the context of a supratherapeutic dose compared to therapeutic doses. Moreover, the diagnosis of DITP was complicated by a broad differential including other causes of thrombocytopenia in critically ill patients.

Calcium channel blockers remain an important but rare cause of thrombocytopenia that must be considered in the diagnostic work-up in order to permit timely discontinuation of the offending agent, preventing severe bleeding and death. Our case is unique because it highlights the fact that the literature in regard to calcium channel blocker overdose is sparse. The actual toxic dose and recommendations for treatment of overdose are unclear, and supportive care remains the best management. We hope to add to the literature by sharing our patient's presentation and management.
